# EXPLORING WISDOM IN PERSONS AGING WITH DISABILITY

**DOI:** 10.1093/geroni/igad104.0128

**Published:** 2023-12-21

**Authors:** Hye Soo Lee, Maya Dye, Wendy Rogers

**Affiliations:** University of Illinois--Urbana-Champaign, Champaign, Illinois, United States; University of Illinois Urbana-Champaign, Champaign, Illinois, United States; University of Illinois Urbana-Champaign, Champaign, Illinois, United States

## Abstract

Wisdom can benefit one’s well-being and lead to a more fulfilling life. One pathway is using wisdom as a resource for coping with adversities in life. At the same time, wisdom can be fostered from experiencing adversities in life. The TechSAge ACCESS interview study explored the challenges and coping strategies of individuals aging with disability (i.e., hearing, vision, and mobility), providing us the opportunity to investigate the wisdom fostered from participants’ lived experiences. Studying wisdom in a population with disability has at least two practical implications for the intersectional field of aging, disability, and technology. First, we can gain and disseminate the knowledge regarding how to cope with aging with disability, which would benefit individuals with similar disability and their care networks. Second, we can utilize participants’ wisdom to guide our research. Focusing on their strengths and coping strategies instead of their weaknesses would offer us additional ways to support individuals aging with disability. There are specific merits to utilizing the ACCESS data for wisdom research. The sample of individuals aging with disability allows us to explore wisdom that manifested as an interaction between a person and a situation. We are exploring participants’ answer to the question regarding the advice they would give to others with similar disability as a wisdom assessment task. This is a contribution to the wisdom literature as it is rare to be able to administer assessment tasks that are relevant to participants’ lives and interests.

